# Red meat and chicken consumption and its association with high blood pressure and obesity in South Korean children and adolescents: a cross-sectional analysis of KSHES, 2011–2015

**DOI:** 10.1186/s12937-017-0252-7

**Published:** 2017-05-22

**Authors:** Geum Hee Kim, Sang Won Shin, Juneyoung Lee, Jun Hyun Hwang, Soon-Woo Park, Jin Soo Moon, Hyun Jung Kim, Hyeong Sik Ahn

**Affiliations:** 1 Department of School Health Education, Sanggye High School, 432, Nohaero Nowon-gu, Seoul, 01761 Republic of Korea; 2 Department of Medicine, College of Medicine, Korea University, 73, Inchon-ro, Seongbuk-gu, Seoul, 02841 Republic of Korea; 3 Department of Biostatistics, College of Medicine, Korea University, 73, Inchon-ro, Seongbuk-gu, Seoul, 02841 Republic of Korea; 4 Department of Preventive Medicine, Catholic University of Daegu School of Medicine, 33, Duryugongwon-ro 17-gil, Nam-gu, Daegu, 42472 Republic of Korea; 5 Division of Pediatric Gastroenterolgy, Hepatology and Nutrition, Department of Pediatrics, Seoul National University, Children’s Hospital, 101 Daehakro, Jongno-gu, Seoul, 03080 Republic of Korea; 6 Department of Preventive Medicine, College of Medicine, Korea University, 73, Inchon-ro, Seongbuk-gu, Seoul, 02841 Republic of Korea

**Keywords:** Meat consumption, High blood pressure, Obesity, Children and adolescents

## Abstract

**Background:**

The impact of meat consumption on high blood pressure (HBP) and obesity in children and adolescents is a subject of debate. The aim of this study was thus to evaluate the association between meat consumption and both HBP and obesity in this group.

**Methods:**

We performed a cross-sectional analysis using nationally representative samples of children and adolescents aged 9, 12, and 15 years old (*n* = 136,739) who were included in the Korea School Health Examination Survey (KSHES) for the 2011–2015 period. Multiple linear and logistic regression analysis was used to determine the factors influencing systolic blood pressure (SBP), diastolic blood pressure (DBP), and body mass index (BMI, kg/m^2^) levels, and to test the strength of these relationships.

**Results:**

Adjusted for covariates, 6.3% of those subjects who consumed >5 servings of meat (including beef, pork, and chicken) per week were obese, compared with 9.1% of the subjects who consumed <1 serving of meat/wk (obesity adjusted odds ratio [OR]: 1.44; 95% confidence interval [CI]: 1.21–1.70; *P* ≤0.001). Those who consumed <1 serving of meat/wk had an HBP prevalence of 8.2%, compared with 7.2% for subjects who consumed >5 servings of meat/wk (systolic HBP adjusted OR: 1.30; 95% CI: 1.05–1.62; *P* ≤0.01, diastolic HBP adjusted OR: 1.25; 95% CI: 1.02–1.54; *P* <0.05). Obese subjects were estimated to have a higher SBP (*β* = 7.497, P < 0.001) and DBP (*β* = 4.123, P <0.001) than subjects who had no excess weight. Compared to subjects who consumed >5 servings of meat/wk, those who consumed <3 servings of meat/wk had a higher SBP (*β* = 0.574, *P <*0.001) and DBP (*β* = 0.376, *P* = 0.003) after adjusting for BMI. The intake of milk, fruit, and vegetables was not associated with either SBP or DBP (*P >*0.05). In contrast, BMI was significantly associated with milk, fruits, and vegetables (*P <*0.01).

**Conclusions:**

Among children and adolescents, a higher level of meat consumption was associated with lower SBP, DBP, and BMI, and greater height, suggesting that consuming an appropriate amount of meat is important for healthy growth at a young age.

## Background

The prevalence of high blood pressure (HBP) and obesity in children and adolescents has increased in the past years [1–8]. To lower blood pressure, several lifestyle changes are recommended, such as weight loss, exercise, and following a healthy diet [9]. An individual’s diet is one of the main modifiable risk factors in the development of HBP [2, 9–16] and, in particular meat intake may play a role. On the one hand, many studies have found that the consumption of red meat and processed meat is associated with an increased risk of cardiovascular disease [17, 18]. On the other hand, meat and animal products are also important sources of protein, vitamins, minerals, and micronutrients that are vital to human health [19]. In fact, *The Dietary Guidelines for Americans* recommend the consumption of lean meat as part of a healthy diet and suggest there may be better ways to incorporate meat into healthy dietary patterns [20, 21]. In addition, recent research has shed light on the crucial role red meat has played in human evolution [15, 16, 19, 20, 22–26]. However, the association between nutrition and cardiovascular health [27] and the impact of meat consumption on HBP and obesity in both adults and children remain subjects of debate [28].

Therefore, the purpose of the present study was to examine the association between meat consumption and both high blood pressure and obesity in a sample of South Korean children and adolescents using a large, nationally representative sample.

## Methods

### Data source and population

We used 5 years of cross-sectional data from the nationally representative Korea School Health Examination Survey (KSHES, 2011–2015; 1,761 schools; *n* = 136,739 students). The Office of Health Statistics collated data from three consecutive 3-year cycles of the KSHES (2009–2011, 2012–2014, and 2015–2017), collecting the data after obtaining written informed consent from the students and their parents. The KSHES is conducted every year by the Ministry of Education in accordance with various laws, including the School Health Act (Article 7), the Standards on Determination and Delivery of School Health Examination Results (Ministry of Education Notice No. 2010–7) for 17 cities and provinces in South Korea, and Guidelines on Electronic Processing and Management of Student Health Records (Ministry of Education Order No. 125). The results of the survey are released as official statistics by the government (Approval No. 11202) [29, 30].

The KSHES includes a stratified, multistage cluster probability sample of students in grades 1–12 who attend public school [29, 30]. For the years used in this study, the first stage of sampling divided administrative districts into 42 to 45 strata depending on their size. The second stage extracted data from 742 to 764 schools, while the third stage extracted data from the first class of each grade. The students belonging to the classes selected in the third stage formed the final sample. Under the KSHES, students are required to visit the hospital for health examinations in grades 1, 4, 7, and 10. These students must visit medical institutions between April and July to receive their health examination, which includes physical development measurements. Therefore, our study sample included students who were in grade 4 (aged 9 years, *n* = 36,733), grade 7 (aged 12 years, *n* = 49,280) and grade 10 (aged 15 years, *n* = 50,726), giving a total of 136,739 students from 1,761 schools. All of the anthropometric and BP measurements included in our study were taken at a hospital.

### Assessment of blood pressure

Blood pressure (BP, mmHg) was measured using an automated digital BP monitor (oscillometric devices models, OMRON MX3). All BP measurements were taken after the subjects had rested quietly in a sitting position for 5 min. The right upper arm was placed at chest level, with the cuff covering at least two-thirds of the length of the arm. Blood pressure was measured using the right arm with an automated upper arm blood pressure monitor with an appropriate cuff size for a child’s arm. Two BP readings were taken 5 min apart. Measures obtained by oscillometric devices that exceeded the 90th percentile were repeated with auscultation [31], the first BP reading discarded and the average of the subsequent measurements taken. The mean of the three SBPs and that of the three DBPs were calculated. Age-, sex-, and height-specific blood pressure cut-offs in Korean children and adolescents were used to define pre-hypertension and hypertension [32]. Using this definition, high blood pressure (HBP) was defined as a systolic BP (SBP) and/or a diastolic BP (DBP) above the 95th percentile for age, sex, and height.

### Assessment of body mass index

Height and weight were measured with standardized procedures, as previously described (KSHES Questionnaires) [29, 30]. Height was measured using a tool consisting of an upright measuring rod and a manual horizontal plate. The subjects were asked to remove their shoes and stand with their heels and buttocks touching the upright rod while resting their arms naturally by their sides. The readings were recorded in 0.1 cm units. Body weight was measured in kilograms with the subjects in an upright position without shoes, in minimal clothing, using a nationally certified, standardized digital scale (CAS, DB-150A). Body Mass Index (BMI) is defined as weight (kg) divided by the square of height (m^2^) [33]. Subjects were grouped into three categories of BMI: no excess weight, overweight, and obesity. Overweight was defined as 85th ≤ BMI < 95th percentile and obesity was defined as BMI ≥ 95th percentile (separately for each age and sex) according to the cutoff points of 2007 Korean growth charts [34]. Children and adolescents with a BMI below the 85th percentile were defined as having no excess weight.

### Assessment of dietary variables

The health behavior survey, which was conducted using a standardized questionnaire produced for the KSHES, and which was filled out at the participating hospitals, consisted of 28 questions that assessed 12 aspects of the respondents’ dietary intake and physical activity. Students were asked to report how many times per week they consumed 5 categories of food: (1) meat, including beef, pork, and chicken, (2) dairy products, including milk, (3) fruit, (4) vegetables, and (5) breakfast. Response options were “daily,” “3 to 5 days per week,” “1 to 2 days per week,” and “never” [29, 30].

### Assessment of covariates

We selected factors associated with HBP based on the current literature [2, 35–37]. Because geographic, socio-economic, racial, and ethnic differences have been found in the HBP of children, adolescents, and adults in a number of studies (27, 30, 31), our study adjusted for place of residence in terms of the 17 cities and provinces in South Korea covered by the survey. These areas were divided into two categories: urban (large, small, and medium-sized cities) and rural (islands, isolated areas, countryside districts, and small towns).

Subjects were also asked to report their physical activity and game/internet use. The survey asked “How many times a week do you exercise to the degree that you have shortness of breath and are sweaty?” with choices of “never,” “1 to 2 days per week,” “3 to 4 days per week,” and “5 to 6 days per week” and “Do you use the internet or play games for more than 2 h per day?” with “yes” or “no” as the two options.

### Statistical analysis

The analysis was done using Statistical Analysis System (SAS version 9.4) and the IBM Statistical Package of Social Sciencess (SPSS version 21) software to deal with the complex sampling designs. We merged the available data from 2011 to 2015 and the analyses were adjusted for the strata, primary sampling units, and probability weights used in the complex sampling design of the KSHES. The demographics of the subjects were recorded as an unweighted sample size and a weighted percentage using composite sample descriptive statistics. For multivariable analysis, prevalence estimates and means were calculated for all variables and data sets. An analysis of variance (ANOVA) was conducted on continuous variables (height and BMI), and categorical variables were expressed in frequency percentages and compared using chi-squared tests. Estimates of proportions, means (standard error of the mean, SEM), and percentiles were calculated. Multivariable analysis was conducted based on variables considered significant (*P* < 0.05) after univariate analysis. A multiple linear regression analysis was performed in order to identify variables that affect BP after controlling for sex, age, height, region, BMI, dietary patterns, and physical activity. Adjusted coefficients of determination (adjusted *R* ^2^), and the *β* coefficient for the associations were estimated using multivariate linear regression models. In addition, adjusted odds ratios (ORs) with 95% confidence intervals (CIs) for the associations were estimated using binary logistic regression models. We used logistic regressions to model the associations between four categories of meat consumption and two outcomes: HBP and obesity. In Model 1, we adjusted for sociodemographic covariates, including sex (girl or boy), age (9, 12, and 15 years), height (centimeter), and region (rural or urban, and 17 cities and provinces in South Korea). In Model 2, we adjusted for BMI (kg/m^2^) when modelling HBP and for BP (mm Hg) when modelling obesity. In Model 3, we further adjustd for dietary variables, including milk (quartiles), fruit (quartiles), vegetable (quartiles), and breakfast (quartiles). In Model 4, we further adjusted for physical activity and game/internet use.

## Results

### Characteristics according to the frequency of meat consumption

Of the 136,739 subjects, 9,918 (7.7%) had high systolic and/or diastolic BP and 9,528 (7.6%) were obese. Of those subjects who consumed meat (including beef, pork, and chicken) every day (intake >5 servings/wk), 7.2% had HBP, compared to 8.2% for those who consumed little meat (intake <1 servings/wk). In addition, 6.3% of those subjects who consumed meat every day were obese, compared to 9.1% in subjects who consumed little meat (Table 1).

**Table 1:** Subject characteristics according to frequency of meat consumption per week

Merged 2011-2015^a^		Consumption of meat per week				
Variables		<1	1–2	3–5	>5	
		*n* (%)^c^ or Mean (SEM)				*P*- value^b^
Subjects *n* (%)^c^	136,739 (100)	4,801 (3.4)	70,193 (51.8)	48,538 (37.1)	10,165 (7.7)	<0.001
Girls	64,760 (47.7)	2,428 (3.6)	33,992 (53.1)	22,656 (36.2)	4,456 (7.1)	<0.001
Boys	71,979 (52.3)	2,373 (3.1)	36,201 (50.7)	25,882 (37.9)	5,709 (8.3)	<0.001
HBP *n* (%)^c^	9,918 (7.7)	387 (8.2)	5,190 (7.7)	3,610 (7.8)	731 (7.2)	<0.001
Systolic HBP	6,075 (4.8)	259 (5.6)	3,287 (5.0)	2,036 (4.6)	405 (4.3)	<0.001
Diastolic HBP	6,704 (5.3)	245 (5.4)	3,302 (5.2)	2,458 (5.4)	520 (5.1)	<0.001
Obesity, *n* (%)^c^	9,528 (7.6)	399 (9.1)	5,337 (8.2)	3,203 (6.9)	589 (6.3)	<0.001
9 years	Anthropometric characteristics					
*n* = 36,733	Height, cm	138.80 (0.2)	138.90 (0.1)	138.61 (0.1)	139.10 (0.3)	0.007
	BMI, kg/m^2^	18.53 (0.1)	18.46 (0.0)	18.34 (0.0)	18.29 (0.1)	<0.001
	Obesity, *n* (%)^c^	138 (9.8)	1,736 (7.9)	694 (7.0)	53 (6.2)	0.001
	Blood pressure					
	Systolic BP, mmHg	99.64 (0.4)	99.00 (0.2)	99.13 (0.3)	98.97 (0.6)	0.004
	Diastolic BP, mmHg	60.48 (0.3)	60.22 (0.2)	60.44 (0.2)	60.08 (0.6)	0.114
	HBP, *n* (%)^c^	106 (7.1)	1,576 (6.9)	657 (6.6)	58 (6.4)	<0.001
12 years	Anthropometric characteristics					
*n* = 49,280	Height, cm	156.99 (0.2)	157.28 (0.1)	157.51 (0.1)	157.92 (0.2)	<0.001
	BMI, kg/ m^2^	20.59 (0.1)	20.48 (0.0)	20.14 (0.0)	20.13 (0.1)	<0.001
	Obesity, *n* (%^c^	163 (8.5)	2,117 (8.1)	953 (6.0)	128 (5.5)	0.001
	Blood pressure					
	Systolic BP, mmHg	105.08 (0.4)	105.22 (0.2)	105.17 (0.2)	105.33 (0.3)	0.327
	Diastolic BP, mmHg	63.32 (0.2)	63.33 (0.1)	63.39 (0.1)	63.20 (0.2)	0.204
	HBP, *n* (%)^c^	172 (7.9)	2,023 (7.7)	1,111 (7.4)	158 (6.6)	<0.001
15 years	Anthropometric characteristics					
*n* = 50,726	Height, cm	165.72 (0.4)	166.31 (0.2)	166.38 (0.2)	166.71 (0.2)	0.007
	BMI, kg/ m^2^	21.96 (0.1)	21.90 (0.0)	21.74 (0.0)	21.64 (0.0)	<0.001
	Obesity, *n* (%)^c^	98 (9.2)	1,484 (8.6)	1,556 (7.4)	408 (6.6)	0.001
	Blood pressure					
	Systolic BP, mmHg	109.21 (0.5)	109.29 (0.2)	109.00 (0.2)	108.71 (0.3)	0.004
	Diastolic BP, mmHg	65.55 (0.3)	65.70 (0.1)	65.55 (0.1)	65.28 (0.2)	0.114
	HBP, *n* (%)^c^	109 (10.2)	1,591 (8.8)	1,842 (8.7)	515 (7.5)	<0.001

^a^Cross-sectional data from the Korea School Health Examination Survey (2011–2015, 1761 schools)

^b^ *P*-value, using chi-squared test for *n* (%), ANOVA for mean (standard error of the mean, SEM)

^c^Unweighted sample size and weighted percentage. In addition, sample sizes vary because of missing data

**Fig. 1:**
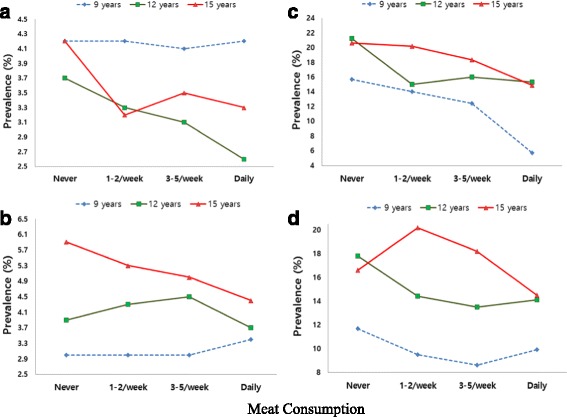
Prevalence of high blood pressure according to the frequency of meat consumption. By age (9, 12, and 15 years), the weighted percentage of systolic and diastolic high blood pressure (HBP) in subjects with no excess weight (Panel **a**: systolic HBP, Panel **b**: diastolic HBP, *n* = 116,475) and with obesity (Panel **c**: systolic HBP, Panel **d**: diastolic HBP, *n* = 9,746) according to the frequency of meat consumption (serving/wk) after adjustment for sex and height. Overall trends are estimated using chi-squared tests. Korea School Health Examination Survey, 2011–2015

The weighted percentage of systolic and diastolic HBP in subjects with no excess weight (*n* = 116,475) and with obesity (*n* = 9,746) according to the frequency of meat consumption is shown in Fig. 1 by age. Among 15-year-old adolescents with no excess weight, prevalence of systolic HBP was 4.2% and diastolic HBP 5.9% in those who consumed little meat, whereas it was 3.3% and 4.4% respectively in those who consumed meat every day (Fig. 1a and b). Meanwhile, among 15-year-old obese adolescents, prevalence of systolic HBP was 20.6% and diastolic HBP 16.6% in those who consumed little meat, whereas it was 14.9% and 14.5% respectively in those who consumed meat every day (Fig. 1c and d).

### Factors associated with BMI

Table 2 presents factors associated with BMI, including place of residence, which were simultaneously adjusted using multiple linear regression (girls: *R* ^2^ = 0.231; boys: *R* ^2^ = 0.191). Subjects with systolic HBP were estimated to have a higher BMI (girls: *β* =1.878; boys: *β* = 2.599, *P* <0.001) than normotensive subjects. Compared to subjects who ate more than 5 servings of meat per week, those who consumed less than 3 had a higher BMI (girls: *β* = 0.441, *P* <0.001; boys: *β* = 0.176, *P* =0.004). Intake of milk and other dairy products, fruit, vegetables, and breakfast also significantly affected BMI (Wald test *P* <0.05; Table 2).

**Table 2:** Adjusted coefficients of regression parameters for BMI in relation to meat consumption

Merged 2011-2015^a^ (*n* = 136,739) Variables^d,e^	Body Mass Index, kg/m^2^							
	Girls^b^				Boys^b^			
	*β*	SEM	*P-* value^c^	*R* ^2^	*β*	SEM	*P-* value^c^	*R* ^2^
Adjusted R^2^				0.231				0.191
Age (years)								
12 y	0.442	0.06	<0.001		−0.665	0.07	<0.001	
15 y	1.557	0.08	<0.001		−0.847	0.10	<0.001	
Height (cm)	0.099	0.01	<0.001		0.121	0.01	<0.001	
Areas								
Rural	−0.084	0.05	0.095		−0.071	0.04	0.130	
Blood pressure (mmHg)	0.795	0.01	<0.001		0.116	0.01	<0.001	
Systolic hypertension	1.878	0.11	<0.001		2.599	0.10	<0.001	
Diastolic hypertension	0.728	0.11	<0.001		1.314	0.11	<0.001	
Dietary patterns (<3/wk)								
Meat	0.441	0.05	<0.001		0.176	0.06	0.004	
Milk	−0.296	0.04	<0.001		−0.302	0.04	<0.001	
Fruits	0.434	0.04	<0.001		0.222	0.04	<0.001	
Vegetable	−0.100	0.04	0.018		−0.208	0.04	<0.001	
Breakfast	0.327	0.05	<0.001		0.148	0.06	0.021	
Physical and leisure activities								
Exercise (<3/wk)	−0.329	0.04	<0.001		0.056	0.03	0.097	
Internet or games (≥2 hr/d)	0.271	0.04	<0.001		0.263	0.04	<0.001	

^a^Cross-sectional data from the Korea School Health Examination Survey (2011–2015, 1761 schools)

^b^Outcome variables: Body mass index (BMI)

^c^ *P-*value of Wald’s test; *β*, coefficient of regression parameters; SEM, standard error of the mean; *R* ^2^, coefficient of determination

^d^All variables in the left column of the table are explanatory variables, and their associations (including place of residence, 17 cities and provinces in South Korea) with BMI were simultaneously adjusted using multiple linear regression

^e^Reference group: Aged 9 y = 0; Urban areas = 0; Dietary patterns, food intake > 5 servings/wk = 0; Exercise ≥ 5/wk = 0; Internet or games < 2 hr/d = 0

### Factors associated with blood pressure

Factors associated with BP, including place of residence, were simultaneously adjusted using multiple linear regression (SBP: *R* ^2^ = 0.260; DBP: *R* ^2^ = 0.162) and the results are reported in Table 3. Obese children and adolescents were estimated to have a higher SBP (+7.49 mmHg; *β* =7.497, *P* <0.001) and DBP (+4.12 mmHg; *β* =4.123, *P* <0.001) than children and adolescents who had no excess weight. Compared to subjects who ate more than 5 servings of meat a week, those who consumed less than 3 had a higher SBP (+0.57 mmHg; *β* =0.574, *P <*0.001) and DBP (+0.37 mmHg; *β* =0.376, *P* =0.003). Those who consumed less than 3 servings of breakfast a week also had a higher DBP (DBP: *β* =0.202, *P* =0.035) than those who consumed breakfast daily. However, we found that the intake of milk (SBP: *β* = −0.129, *P =*0.264; DBP: *β* =0.023, *P* =0.767), fruit (SBP: *β* =0.143, *P =*0.189; DBP: *β* = −0.128, *P* = 0.098), and vegetables (SBP: *β* =0.044, *P =*0.661; DBP: *β* =0.016, *P* =0.817) were not associated with either SBP or DBP (Table 3).

**Table 3:** Adjusted coefficients of regression parameters for blood pressure in relation to meat consumption

Merged 2011-2015^a^ (*n* = 136,739) Variables^d,e^	Blood pressure (BP), mmHg							
	Systolic BP^b^				Diastolic BP^b^			
	*β*	SEM	*P-* value^c^	*R* ^2^	*β*	SEM	*P-* value^c^	*R* ^2^
Adjusted R^2^				0.260				0.162
Sex								
Boys	1.137	0.11	<0.001		0.467	0.07	<0.001	
Age (years)								
12 y	1.339	0.29	<0.001		0.896	0.20	<0.001	
15 y	2.964	0.33	<0.001		2.111	0.22	<0.001	
Height (cm)	0.260	0.01	<0.001		0.103	0.01	<0.001	
Areas								
Rural	−0.089	0.28	0.757		−0.003	0.19	0.987	
BMI (kg/m^2^)	0.795	0.01	<0.001		0.116	0.01	<0.001	
Overweight	4.452	0.13	<0.001		2.265	0.09	<0.001	
Obesity	7.497	0.16	<0.001		4.123	0.10	<0.001	
Dietary patterns (<3/wk)								
Meat	0.574	0.16	<0.001		0.376	0.12	0.003	
Milk	−0.129	0.17	0.264		0.023	0.07	0.767	
Fruits	0.143	0.10	0.189		−0.128	0.07	0.098	
Vegetable	0.044	0.09	0.661		0.016	0.07	0.817	
Breakfast	0.240	0.13	0.065		0.202	0.09	0.035	
Physical and leisure activities								
Exercise (<3/wk)	0.058	0.10	0.567		0.134	0.06	0.047	
Internet or games (≥2 hr/d)	0.077	0.11	0.490		0.006	0.07	0.930	

^a^Cross-sectional data from the Korea School Health Examination Survey (2011–2015, 1761 schools)

^b^Outcome variables: SBP or DBP

^c^ *P-*value of Wald’s test; *β*, coefficient of regression parameters; SEM, standard error of the mean; *R* ^2^, coefficient of determination

^d^All variables in the left column of the table were explanatory variables, and their associations (including place of residence, 17 cities and provinces in South Korea) with SBP or DBP were simultaneously adjusted using multiple linear regression

^e^Reference group: Girls = 0; Aged 9 y = 0; Body mass index (BMI), No excess weight = 0; Urban areas = 0; Dietary patterns, food intake > 5 servings/wk = 0; Exercise ≥ 5/wk = 0; Internet or games < 2 hr/d = 0

### Associations between meat consumption, obesity, and high blood pressure

Table 4 displays the results of the multivariate logistic regression. There were significantly increased odds of HBP and obesity in subjects who consumed less than 1 serving of meat a week, compared with those who consumed more than 5. In a basic Model 1 (adjusted for sociodemographic variables), compared with those who consumed meat more than 5 times a week, subjects who consumed less than 1 serving were significantly more likely to have a higher prevalence of HBP and obesity (systolic HBP: adjusted odds ratio [OR] 1.43, 5% confidence interval [CI] 1.16–1.75; diastolic HBP: OR 1.37, 95% CI 1.11–1.69; obesity: OR 1.62; 95% CI: 1.39–1.91). In Model 2 (further adjusted for BP or BMI when modelling obesity and HBP, respectively), Subjects who consumed meat at a rate of <1 serving/wk had significantly increased odds of HBP and obesity compared with those who consumed >5 servings/wk. In contrast to the first model, the second model finds an association between HBP and obesity in children and adolescents. The final model indicated that the consumption of less than 1 serving of meat per week was associated with a higher prevalence of HBP (OR for systolic: 1.30; 95% CI: 1.05–1.62) and obesity (OR: 1.44; 95% CI: 1.21–1.70), compared with the consumption of meat at a rate of more than 5 servings a week.

**Table 4:** Association between meat consumption and both obesity and high blood pressure in South Korean children and adolescents

*n* = 136,739 Aged 9, 12, and 15 y	Consumption of meat (servings/wk)^c^			
	<1	1–2	3–5	>5
Obesity^a^, ORs (95% CI)^b^				
Model 1, multivariable^d^	1.62*** (1.39–1.91)	1.42*** (1.28–1.59)	1.16** (1.04–1.29)	Reference
Model 2, model 1 plus BP^e^	1.50*** (1.27–1.76)	1.36*** (1.21–1.52)	1.13* (1.01–1.26)	Reference
Model 3, model 2 plus dietary patterns^f^	1.43*** (1.21–1.69)	1.30*** (1.16–1.46)	1.12* (1.00–1.25)	Reference
Model 4, model 3 plus activities^g^	1.44*** (1.21–1.70)	1.30*** (1.16–1.46)	1.12* (1.00–1.25)	Reference
Systolic HBP^a^, ORs (95% CI)^b^				
Model 1, multivariable^d^	1.43*** (1.16–1.75)	1.23** (1.08–1.41)	1.10 (0.96–1.26)	Reference
Model 2, model 1 plus BMI^e^	1.29** (1.04–1.60)	1.14 (0.99–1.31)	1.06 (0.92–1.22)	Reference
Model 3, model 2 plus dietary patterns^f^	1.31** (1.06–1.62)	1.15* (1.00–1.32)	1.06 (0.92–1.22)	Reference
Model 4, model 3 plus activities^g^	1.30** (1.05–1.62)	1.15* (1.00–1.31)	1.05 (0.92–1.21)	Reference
Diastolic HBP^a^, ORs (95% CI)^b^				
Model 1, multivariable^d^	1.37** (1.11–1.69)	1.27*** (1.11–1.45)	1.19** (1.04–1.36)	Reference
Model 2, model 1 plus BMI^e^	1.28* (1.03–1.59)	1.20* (1.04–1.38)	1.16* (1.16–1.33)	Reference
Model 3, model 2 plus dietary patterns^f^	1.26* (1.02–1.55)	1.20** (1.04–1.38)	1.17* (1.02–1.33)	Reference
Model 4, model 3 plus activities^g^	1.25* (1.02–1.54)	1.19** (1.04–1.37)	1.16* (1.01–1.33)	Reference

^a^Outcome variables: High blood pressure (HBP) or Obesity

^b^*P < 0.05; **P ≤ 0.01; ***P ≤ 0.001, using binary multiple logistic regression analysis for adjusted odds ratios (ORs) and 95% confidence interval (CI)

^c^Explanatory variable: meat consumption (servings per wk)

^d^Adjusted for sex, age, height, and regions (17 cities and provinces in South Korea)

^e^Additional adjustment for BP (mm Hg) when modelling obesity and for BMI (kg/m^2^) when modelling HBP

^f^Additional adjustment for milk (quartiles), fruit (quartiles), vegetable (quartiles), and breakfast (quartiles)

^g^Additional adjustment for physical activities and internet/games use

In summary, a statistically significant association was found at all levels of adjustment. The less meat subjects ate, the greater the likelihood of exhibiting HBP and obesity (*P* <0.05). In this analysis, the ORs of HBP and obesity in children and adolescents fell as the frequency of meat consumption increased after adjustment for covariates related to HBP.

## Discussion

In our study involving 136,739 children and adolescents of 9, 12, and 15 years of age who participated in the Korea School Health Examination Survey from 2011 through 2015, a higher level of red meat and chicken consumption was associated with lower prevalence of HBP and obesity after adjusting for potential confounding variables. In addition, a higher prevalence of HBP was associated with obesity.

HBP increases the risk of long-term cardiovascular disease, as well as the risk of health complications and death in adulthood [38]. Therefore, there is a need to prevent HBP and obesity in childhood. In this study, 7.7% of the children and adolescents in the survey had HBP. A previous study has reported that the prevalence of HBP in adolescents across a number of countries was 11.2% (13% for boys and 9.6% for girls) [39], meaning South Korea adolescents have a relatively low HBP rate. In the present study, BP varied with age, height, BMI, and gender, meaning these variables are likely to influence the risk of HBP. For example, boys had a higher prevalence of HBP than did girls. These results were consistent with those of previous studies [12, 35, 40, 41].

We found that children and adolescents who rarely consumed meat had a higher rate of both HBP and obesity compared with those who ate meat daily. Our study demonstrated that obesity in children and adolescents is associated with an increased prevalence of HBP. Even when our analysis controlled for BMI and other potential confounding factors, we found that HBP was more common in those children and adolescents with lower meat consumption.

Red meat and chicken provide a rich source of protein of high biological value and essential nutrients when included as part of a healthy and varied diet [22]. A recent report from the Beef in an Optimal Lean Diet (BOLD) study showed that an increase in lean-beef consumption while controlling saturated fatty acid intake (6% of total calories) in the context of a heart-healthy diet was associated with significant decreases in total cholesterol and low-density lipoprotein cholesterol (LDL-C) in healthy men and women (30–65 years of age) with elevated LDL-C concentrations [16]. In the BOLD study, SBP decreased by 4 mm Hg after the consumption of a Dietary Approaches to Stop Hypertension (DASH) diet that included 153 g of lean beef per day but did not decrease after DASH diets with 113 g or 28 g of lean beef per day [23]. It has also been found that a moderate protein DASH diet including lean beef or lean pork decreased SBP in normotensive individuals [15, 23].

We observed that children and adolescents with a higher frequency of meat consumption per week had lower BP and BMI and were taller. Westerterp-Plantenga et al.[24] have described that dietary protein contributes to the treatment of obesity and metabolic syndrome by acting on the relevant metabolic targets of satiety and energy expenditure in a negative energy balance, thereby preventing the weight-cycling effect. Meat consumption may be a useful component of weight-loss diets because of the satiating effect of its high protein content [22]. To date, previous studies on BP have tended to focus on adults, including the elderly [12, 18]. However, our study focuses on children and adolescents. Children and adolescents, who are at a stage of rapid growth and development, exhibit large differences in BP depending on sex, age, and height and have different nutritional needs than adults do [7]. Therefore, the negative association between meat consumption and HBP risk in our study did not agree with observations reported in previous studies [12, 18], nor the recommendations of the DASH diet, which encourages lower consumption of red meat [15]. In addition, we found that the consumption of dairy and fruit did not significantly affect BP after adjusting for potential confounding factors. A low intake of meat, especially red meat, is recommended to avoid the risk of cancer and metabolic syndrome [42]. However, this advice does not consider the fact that meat provides important nutrients for the health, growth, and development of children and adolescents [25, 43, 44]. Red meat including beef, pork, and lamb has been an important part of the human diet throughout human evolution [22]. In particular, meat is one of the most nutrient-dense sources of protein, providing iron, vitamin B12, other B complex vitamins, zinc, selenium, and phosphorus [20, 25, 43]. Dietary protein also contributes to the improvement of body composition and lipid and lipoprotein profiles, and the treatment of obesity and metabolic syndrome [24, 45]. Despite these advantages, epidemiological studies have linked meat consumption to HBP [25, 43], with red meat consumption in particular commonly considered a risk factor in HBP because of its saturated fat and cholesterol content [18]. However, these studies have many limitations [25], including that the existing studies are on adults and that other mechanisms may take place in children. Although previous epidemiological data have revealed a possible association between meat consumption and the increased risk of cancer and cardiovascular and metabolic disease [17, 19, 42, 46–49], moderate meat consumption of up to ~100 g/day was not associated with increased mortality from ischemic heart disease, stroke, or cardiovascular disease of all types among Japanese men and women [26]. According to the OECD, meat consumption (beef and veal, pork, poultry, and sheep) in Asian countries in 2015 (9.6 kg per person in Korea and 6.7 in Japan) is significantly lower than in North and South America (24.7 kg per person in the United States, 40.4 in Argentina, and 46.4 in Uruguay) [50]. In our study, meat consumption proved to be a significant factor of the human diet in regions, countries, and ethnic groups in which meat consumption is relatively low.

The strengths of our study are as follows. Our study is the first to investigate meat consumption and its association with HBP and obesity among children and adolescents in a large and nationally representative population in Korea. In addition, a valid sample was extracted using stratified multistage cluster sampling and probability sampling, and is thus an accurate representation of the child and adolescent population in Korea. Furthermore, the subjects visited medical institutions, where an automated BP monitor with a high level of reliability was used to measure BP, and data were obtained from laboratory and physical examinations using standardized protocols to minimize the influence of measurement error. Finally, the descriptive statistics from the separate Korea School Health Examination Survey (KSHES) cycles produced similar frequencies, percentages, and averages each year. The results were found to have a Cronbach’s alpha of more than 0.5, which indicates a high degree of replicability.

However, our study had several limitations. First, this cross-sectional study did not allow us to evaluate the temporal relationship between meat consumption and HBP. Because risk factors and HBP are measured at the same time, it is difficult to determine which of the two occurred first, and cause and effect inferences thus could not be made. Second, the subjects visited hospitals in their neighborhood, and some errors may have occurred in BP measurements due to differences in the BP monitoring equipment used. However, the medical institutions in question adhered to the standard protocols of BP measurement as recommended by the KSHES, so the results can be considered reliable because the annual measurements produced similar means for BP. Third, socioeconomic status (SES) can influence both BP and obesity but not taken into account here [8, 51], because SES indicators (e.g., parental education, parental occupation, and family income) are not included in the standardized questionnaire produced for the KSHES. Finally, meat intake (g/day or g/serving) was not included in the question on food intake in the KSHES questionnaire used in this study. In addition, the question about the frequency of meat consumption did not distinguish between the type of meat (red or white, lean or fatty, and processed or unprocessed) or the type of cooking (e.g., fried or boiled). As a result, this study was unable to assess possible differences in BP resulting from the consumption of different types of meat. However, we found a significant decrease in SBP, DBP, and BMI with increasing meat consumption and a resulting positive effect on the growth of children and adolescents.

## Conclusions

In this nationally representative sample of Korean children and adolescents, we found that meat consumption was inversely associated with HBP and obesity. The odds of HBP were greater among children and adolescents who rarely ate meat compared to those who ate meat daily. An adequate meat intake may therefore contribute to a healthier blood pressure and body weight in children and adolescents.

## Abbreviations

95% CI: 95% confidence interval
BMI: Body mass index
DBP: Diastolic blood pressure
HBP: High blood pressure
KSHES: Korea School Health Examination Survey
OR: Odds ratio
SBP: Systolic blood pressure
